# Successful long-term control of poultry red mite (*Dermanyssus gallinae*) infestations in floor-kept laying hens via integrated pest management—a case report

**DOI:** 10.1007/s00436-023-07954-9

**Published:** 2023-08-29

**Authors:** Vanessa Rüster, Alina Kathrin Lückemann, Margareta Wittmann, Christina Strube, Thomas Bartels

**Affiliations:** 1 Institute of Animal Welfare and Animal Husbandry, Friedrich-Loeffler-Institut, Celle, Germany; 2 Institute for Parasitology, Centre for Infection Medicine, University of Veterinary Medicine Hannover, Hannover, Germany; 3 Chamber of Agriculture Lower Saxony, Oldenburg, Germany; 4 Faculty of Agriculture, South Westphalia University of Applied Sciences, Soest, Germany

**Keywords:** Pest control, Acaricide, Fluralaner, Exzolt^®^, Amorphous silica, Fossil Shield^®^, Tube traps

## Abstract

This case report describes the successful control of poultry red mite [PRM] (*Dermanyssus gallinae*) infestations in an experimental laying hen house via a combined use of cleaning and disinfection measure, the preventive application of a synthetic silica-based acaricide and frequent mite monitoring. The high number of PRM in the laying hen house was reduced by 99.8% by treatment with fluralaner (Exzolt^®^, MSD Animal Health Unterschleißheim, Germany; 0.5 mg/kg body weight via drinking water twice, 7 days apart). After the laying hens were removed, the hen house was dry-cleaned, wet-cleaned and disinfected. After drying, synthetic amorphous silica (Fossil Shield^®^ instant white, Bein GmbH, Eiterfeld, Germany) was applied as a preventive measure before the hen house was restocked with pullets for two housing periods of 58 and 52 weeks. Over these periods (i.e. more than 2 years), no PRM was detected during mite monitoring at two-week intervals via tube traps and visual monitoring. This result therefore suggests that the combined use of appropriate chemical and physical prevention measures within an integrated pest management regime can be successfully used for the long-term control of PRM. This could reduce the use of acaricidal drugs, thereby helping maintain their efficacy.

## Introduction

Poultry red mite [PRM] (*Dermanyssus gallinae*) infestations are a substantial problem in laying hen husbandry worldwide. In Germany, it is estimated that 94% of laying hen farms are infested with PRM (Mul 2013). Heavy PRM infestations can lead to significant animal health problems and animal welfare issues as well as economic problems. Depending on the degree of infestation, infested laying hens may show impaired resting behaviour and reduced performance parameters such as lower egg weight and anaemia. In severe cases, infestation can even lead to sudden death (Kilpinen et al. 2005; Flochlay et al. 2017). In addition, PRM can harm laying hens by serving as vectors for various pathogens, e.g. *Salmonella enterica* subsp. *enterica* ser. Gallinarum or avian influenza virus (Sommer et al. 2016; Pugliese et al. 2019). PRM infestations also pose a zoonotic risk, as humans can be affected as off-target hosts, resulting in local skin irritations and even causing avian mite dermatitis (Cafiero et al. 2019).

Monitoring is an important measure to determine the timing of control measures (Lima-Barbero et al. 2020). Continuous mite monitoring can be used to detect an infestation at the early stage, observe the development of the mite population and check the effectiveness of control methods (Meyer-Kühling 2007). In addition, the development of the infestation can be followed by regular mite monitoring (Zenner et al. 2009). The more severe the mite infestation is, the more difficult it is to control and the more serious the consequences for the hens.

Conventional use of chemical acaricides is increasingly limited by the rapid development of acaricide resistance, the risk of residues in eggs and meat and strict legislation. In addition, consumer demand for pesticide-free products is increasing (Mul 2013). This has led to increased research into alternative control methods for PRM in recent years (Maurer et al. 2009; Decru et al. 2020; Lima-Barbero et al. 2020; Alves et al. 2020). There is an urgent need for effective and safe control strategies, i.e. sustainable treatment options and preventive measures (Tomley and Sparagano 2018).

This report describes successful long-term PRM control in floor-kept laying hens via the initial acaricidal treatment of flocks, followed by chemophysical environmental measures and implementation of a PRM monitoring system as parts of an integrated pest management regime.

## Materials and methods

### Animals and husbandry conditions

The affected laying hens were housed in a floor husbandry system at the experimental station of the Institute for Animal Welfare and Animal Husbandry at the Friedrich-Loeffler-Institut, Celle, Germany. The entrance area of the barn was equipped with disinfection baths. Access was also restricted to staff of the experimental station with protective clothing. The hen house was divided into 20 identical compartments, which were separated by metal grids. The compartments, each with a floor area of 4 m^2^, were divided into an upper grid area and a lower litter area, which was covered with dry straw. The upper area of 2 m^2^ contained a suspended round drinker, three resting perches and a group nest with an approach bar. The manure pit was located below. All laying hens were provided unlimited acces to a commercial complete diet, grit and drinking water.

### Management conditions and treatments

**Fig. 1:**
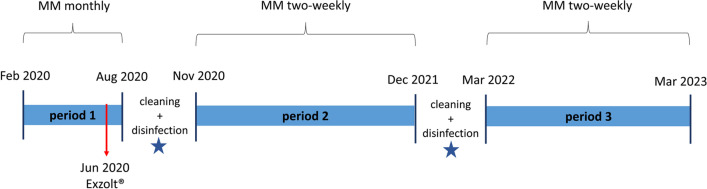
Timeline of the case report: housing periods from February 2020 to March 2023, frequency of the mite monitoring (MM) and measures in the hen houses (star: coating with Fossil Shield^®^ instant white)

After mechanical cleaning, a total of 340 laying hens (Lohmann Brown, age: 40 weeks; stocking density: 4.25 hens/m^2^) were housed for 23 weeks from February to August 2020 (Fig. 1). As a result of a strong natural infestation of PRM, treatment with fluralaner (Exzolt^®^, MSD Animal Health, Unterschleißheim, Germany) was carried out in June 2020. The dosage was calculated according to the package leaflet (0.5 mg/kg body weight twice at a 7-day interval via drinking water; Thomas et al. 2017).

Immediately after the hens left the barn in August 2020, group nests, perches, drinkers and feeders were removed. The litter and contents of manure pits were removed mechanically. The barn equipment was cleaned with a high-pressure cleaner according to good agricultural practice. The barn was wet-cleaned with the alkaline (sodium hydroxide) foam cleaner Menno^®^ Clean (Menno Chemie Vertrieb GmbH, Norderstedt, Germany). After drying, disinfection of the barn and barn equipment was carried out with VENNO^®^ VET 1 super (active ingredient: formic acid, Menno Chemie Vertrieb GmbH, Norderstedt, Germany) and NEOPREDISAN^®^ 135–1 (active ingredient: p-chloro-m-cresol, Menno Chemie Vertrieb GmbH, Norderstedt, Germany). One week before restocking, the hen house and the equipment were disinfected a second time with VENNO^®^ VET 1 super and NEOPREDISAN^®^ 135–1. After drying, an aqueous suspension of synthetic amorphous silica (Fossil Shield^®^ instant white, Bein GmbH, Eiterfeld, Germany) was applied using a pressure sprayer in all compartments of the hen house by a company specialized in PRM control via silicates. The barn floor, walls and equipment, the PRM hiding places such as cracks and crevices, and suspected PRM running paths were coated. Restocking of the hen house was carried out with 500 laying hens of five strains (Lohmann LSL, Lohmann Brown, Bovan, ISA Brown, Dekalb, age: 12 weeks; 6.25 hens/m^2^). The hens were kept for 58 weeks from November 2020 to December 2021. After this period, all barn compartments as well as the barn equipment were treated as described above in the previous service period and restocked again with 500 laying hens of various strains (Lohmann Selected Leghorn, Lohmann Brown, Bovan, ISA Brown, Dekalb, age: 17 weeks) (6.25 hens/m^2^) for 52 weeks from March 2022 to March 2023 (Fig. 1).

### PRM monitoring

For PRM monitoring, corrugated cardboard tube traps were used as described by Rüster et al. (2022). Two tube traps were placed in each compartment, one underneath the approach bar of the group nest and the other below a resting perch. In housing period 1, PRM numbers in the traps were determined once a month from April to July. In contrast, in housing periods 2 and 3, PRM were counted every 2 weeks to enable PRM detection even if a small number was present. The mite traps were kept in the hen house compartments for 48 h. After collection in plastic bags, the traps were immediately frozen at − 20 °C for rapid killing of PRM. Dead mites were then transferred to petri dishes and counted. In addition, mite populations in housing periods 2 and 3 were determined visually using the mite monitoring system of Cox et al. (2009). A scoring system was used to monitor three different locations per barn compartment: inside the layer group nest, underneath the grid and on the perch attachment structures.

## Results and discussion

**Fig. 2:**
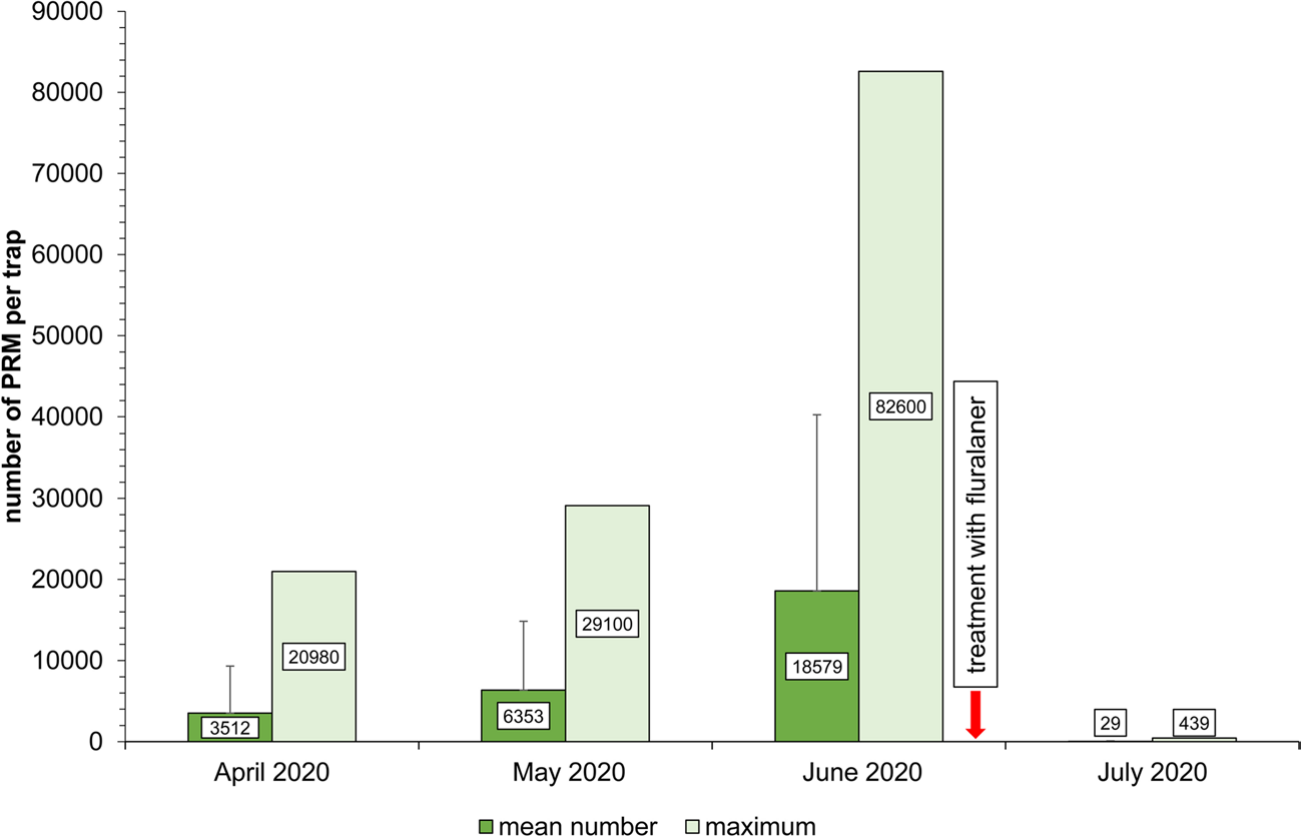
Number of poultry red mites: mean value with standard deviation and maximum number per mite trap in housing period 1 before and after treatment with fluralaner (Exzolt^®^, MSD Animal Health) in June 2020

**Fig. 3:**
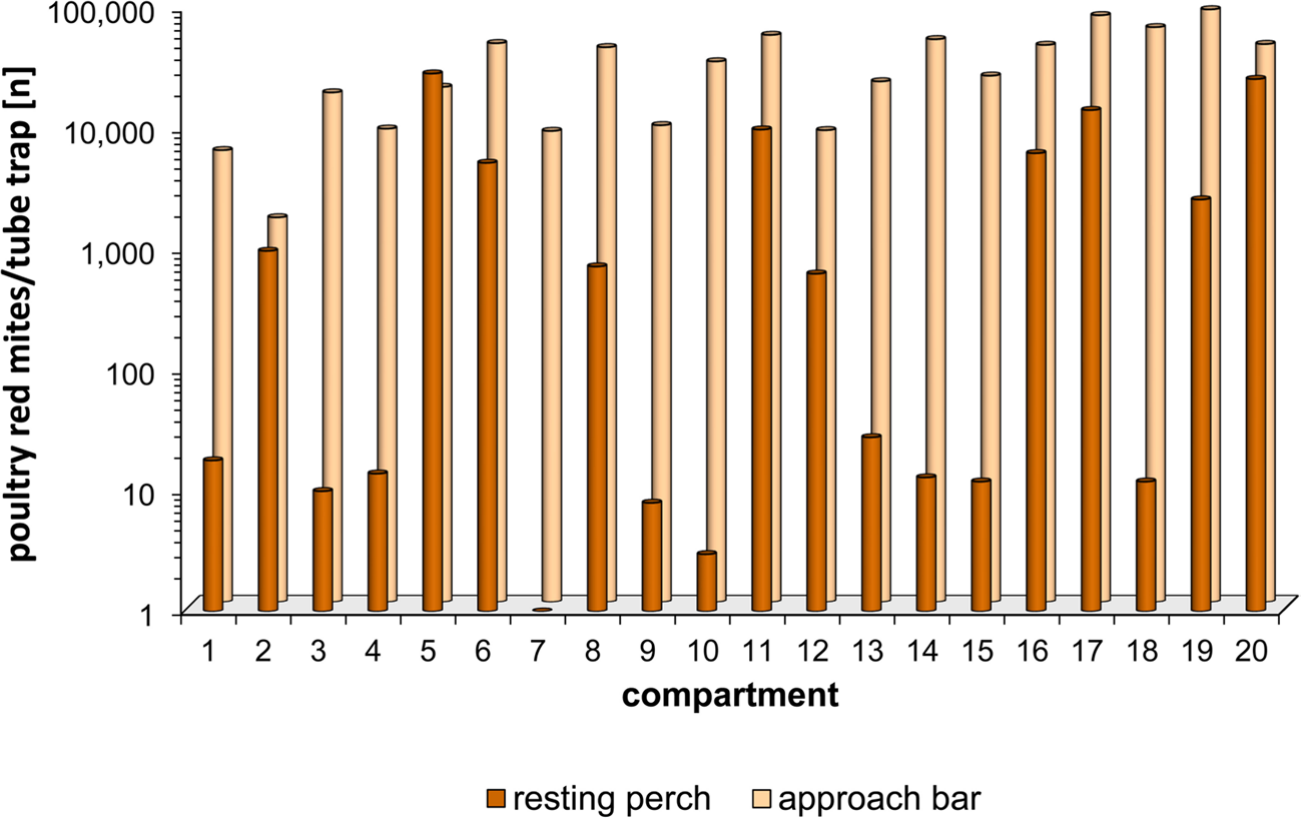
Number of poultry red mites in the 40 individual monitoring tube traps, installed either below the resting perches or the approach bars of the group nests, in June 2020 (housing period 1)

Due to the weather, an increase in the PRM population was seen during mite monitoring in housing period 1, resulting in an average number of more than 18,500 PRMs per trap in the period ending in June 2020 (Fig. 2), with up to 82,600 PRM per mite trap. A total of 743,160 PRM were detected in the 40 monitoring traps installed in the hen house at the sampling time in June 2020. The PRM numbers in the 40 individual traps, of which 20 each were attached to the underside of the resting perches or to the underside of the approach bars of the group nests, are shown in Fig. 3. In response to this strong infestation, acaricidal treatment of the hens with fluralaner (Exzolt^®^, MSD Animal Health) was carried out immediately after mite counting in June 2020 (D0) as described above. It is recommended that the drug be administered twice, 7 days apart, so that postlarval stages that have not taken a first blood meal until after the first administration of the acaricide receive a lethal dose with the second application (Thomas et al. 2017; EMA 2017). In July 2020, 2 weeks after the second treatment (D7), a 99.8% reduction in mite numbers was observed in the monitoring traps (Fig. 2). These findings are consistent with other studies that showed acaricidal efficacies of more than 99% after two applications of fluralaner (Brauneis et al. 2017; Thomas et al. 2017; Petersen et al. 2021). The success of the treatment can be influenced by external factors, e.g. a delay in mite egg or larval development due to low temperatures (Tucci et al. 2008). There is also the possibility that males do not come into contact with the hen blood containing high levels of active agents due to their intermittent feeding behaviour (Nunn 2023).

It is important to note that the number of PRMs determined by trap monitoring only allows limited conclusions to be drawn about the scale of the total mite infestation in the barn. Therefore, mite monitoring results should be used to determine mite population dynamics (Decru et al. 2020). In the context of an IPM, it is recommended to establish thresholds above which appropriate measures should be taken. However, concrete thresholds for characterizing the extent of PRM infestation have not yet been established for PRM (Decru et al. 2020). One reason for this may be that the distribution of PRM in a barn can be very inhomogeneous (cf. Figure 3), which makes it difficult to define threshold values.

No PRM was detected in the monitoring traps in housing periods 2 and 3. Visual monitoring according to the mite monitoring system of Cox et al. (2009) resulted in a score of 0, as no mites were observed during the monitoring period. Mul et al. (2009) emphasized the importance and necessity of thorough cleaning prior to mite control. Wet cleaning followed by a disinfectant with acaricidal ingredients in a still unoccupied barn is recommended (Chauve 1998; Nordenfors and Höglund 2000; Decru et al. 2020). For example, Sommer (2011) and Al Halbouni (2016) described good efficacy efficacy of Neopredisan^®^ 135–1 against all stages of PRM, including mite eggs, and this treatment was used as a disinfectant in the present study. Synthetic amorphous silica has also been described as an efficient preventive measure. It can be applied both in powder form and as an aqueous suspension. Aqueous suspension formulations of amorphous silicate preparations have a longer-lasting effect (Maurer and Perler 2006). Hence, combined treatment with both a disinfectant, such as Neopredisan^®^, and synthetic amorphous silica, such as Fossil Shield^®^ instant white, both of which have acaricidal effects (Schulz et al. 2014; Al Halbouni 2016), resulted in efficient PRM control over a period of more than 2 years.

Consequently, no further acaricidal drug treatment was required for the newly housed laying hens after period 1 due to the implementation of integrated pest management (IPM). IPM is defined as “the careful consideration of all available pest control techniques and subsequent integration of appropriate measures that discourage the development of pest populations and keep pesticides and other interventions to levels that are economically justified and reduce or minimize risks to human health and the environment. IPM emphasizes the growth of a healthy crop with the least possible disruption to agro-ecosystems and encourages natural pest control mechanisms” (FAO 2020). The IPM strategy is divided into eight steps, which are explained in detail in Decru et al. (2020). IPM is a combination of preventive measures, monitoring techniques and alternative control measures with reduced use of chemical acaricides. The aim of IPM is to reduce the use of pesticides and to avoid the development of acaricide resistance to minimize the risks to humans, animals and the environment. Harrington et al. (2011) foresee great potential in an IPM approach as new technologies and techniques for PRM control are adopted for the sustainable control of PRM as an important poultry pest. However, Sparagano et al. (2014) stated that the full potential of integrated pest management in PRM control may not yet have been realized relative to other areas of pest management, such as crop pest control. Similarly, Decru et al. (2020) noted that reports on the practical application of the IPM approach in PRM control are scarce.

In IPM treatment with a chemical acaricide should be used only as a measure of last resort (FAO 2020; Decru et al. 2020). In this case report, the maximum number of PRMs increased approximately threefold (29,100 to 82,600 mites, see Fig. 2) from the sampling date in June to 4 weeks later in July. In our facility, no threshold was set beforehand. Nevertheless, on the basis of the July mite monitoring results, it was decided that physical or alternative measures in the occupied hen house, such as local treatment with synthetic amorphous silica, would not be sufficient to efficiently control the emerged severe PRM infestation. Therefore, fluralaner treatment was chosen and showed a successful reduction in the PRM population. Fluralaner is currently the only acaricidal drug licensed against PRM in laying hens in Germany. Short withdrawal periods of 0 days for eggs and 14 days for meat and offals prevent economic losses for laying hen farms. Its use should be judicious and correctly dosed to avoid the development of acaricide resistance in PRM. This may help to ensure that an effective drug for the treatment of severe PRM infections will continue to be usable in the future.

In June 2020, the average maximum temperature has already reached 30 °C. These conditions were optimal for the development and reproduction of the mite and indicated a further increase in mite numbers (Maurer and Baumgärtner 1992; Nordenfors et al. 1999). The targeted use of fluralaner reduced the PRM population, allowing preventive measures to be applied to take effect. Synthetic amorphous silica is a common biophysical control measure for PRM (Maurer and Perler 2006; Kilpinen and Steenberg 2009; Schulz et al. 2014). They cause lipid loss from the cuticle as well as abrasion of the cuticle, resulting in desiccation (Kilpinen and Steenberg 2009).

Adequate mite monitoring is important to observe PRM population growth and initiate control measures in time to counteract infestation damage (Mul et al 2015), and the combined use of differently acting prevention measures and continued mite monitoring allows the reduced and targeted use of acaricidal drugs. Such a strategic approach and a risk–benefit analysis according to the IPM philosophy (i.e. economic thresholds) help to conserve the efficacy of acaricides in the long term, minimize unacceptable effects on poultry and human health and protect the environment. PRM not only causes animal health problems but also results in massive economic losses (Flochlay et al. 2017). Effective control of PRM through the implementation of IPM can improve the welfare of laying hens and is expected to reduce the substantial economic losses due to performance losses and control costs.

The combined application of different measures as part of a comprehensive IPM strategy could be a solution for future PRM control. The results call for further research to develop an appropriate IPM strategy for PRM control.

## Conclusion

The results of this case report show that PRM in laying hens can be successfully controlled by an IPM programme including mite monitoring, cleaning, disinfection and preventive measures with a physical mode of action. No PRM was detected by mite monitoring for more than 2 years after the application of the IPM strategy. These results suggest that an IPM programme can help to reduce the use of acaricidal drugs. Although this may not completely prevent the development of acaricidal resistance, it will at least delay the development of resistance. Novel methods with physical modes of action, e.g. cold atmospheric pressure plasma (Rüster et al. 2022) or high voltage pulses (Ueno et al. 2023), may also help to reduce the use of chemical acaricides and thus maintain their efficacy in the future.

## Data Availability

Data supporting the reported results are included in the article.
